# Associations of P16^INK4a^ promoter hypermethylation with squamous intra-epithelial lesion, cervical cancer and their clinicopathological features: a meta-analysis

**DOI:** 10.18632/oncotarget.12202

**Published:** 2016-09-22

**Authors:** Ya-di Han, Xue-bin Wang, Ning-hua Cui, Shuai Zhang, Chen Wang, Fang Zheng

**Affiliations:** ^1^ Center for Gene Diagnosis, Zhongnan Hospital of Wuhan University, Wuhan, Hubei, China; ^2^ Department of Clinical Laboratory, Children's Hospital of Zhengzhou, Zhengzhou, Henan, China

**Keywords:** cervical cancer, squamous intra-epithelial lesion, P16^INK4a^ promoter hypermethylation, smoking habit, meta-analysis

## Abstract

To assess the associations of *P16^INK4a^* methylation status with low-grade squamous intra-epithelial lesion (LSIL), high-grade squamous intra-epithelial lesion (HSIL), cervical cancer (CC) and their clinicopathological features, a meta-analysis with 29 eligible studies was conducted. Pooled odds ratios (ORs) with their 95% confidence intervals (CIs) were estimated to assess the strength of the associations. Heterogeneity, sensitivity of pooled results and publication bias were also evaluated. Overall, there was an increasing trend of *P16^INK4a^* hypermethylation rates among LSIL (21.4%), HSIL (30.9%) and CC (35.0%) specimens. *P16^INK4a^* hypermethylation was significantly associated with the increased risk of LSIL, HSIL and CC, with the pooled ORs of 3.26 (95% CI: 1.86-5.71), 5.80 (95% CI: 3.80-8.84) and 12.17 (95% CI: 5.86-25.27), respectively. A significant association was also found between *P16^INK4a^* hypermethylation and smoking habit (OR = 3.88, 95% CI: 2.13-7.08). Taken together, meta-analysis results support *P16^INK4a^* hypermethylation as an epigenetic marker for the progression of cervical carcinogenesis.

## INTRODUCTION

Cervical cancer (CC) is one of the most common gynecologic cancers worldwide [1], with an estimated 527,600 new cases and 265,700 deaths each year [2]. The development of CC is considered as a continuous process from normal epithelium to squamous intra-epithelial lesion (SIL) and ultimately to invasive carcinoma [3]. SIL, the precursor lesions of CC, can be further divided into low-grade SIL (LSIL) and high-grade SIL (HSIL) depending on the risk of cancer progression [4]. Although infection with human papillomavirus (HPV) is a widely accepted risk factor for SIL and CC [5], the evidence that only a small subset of HPV-induced lesions progress to CC [6], suggests that HPV infection is essential but insufficient for cervical carcinogenesis [4].

DNA hypermethylation, the major epigenetic event in humans, can occur at CPG islands within promoter regions of tumor suppressor genes (TSGs), and consequently silence the TSGs' transcription [7]. *P16^INK4a^* gene, a well known TSG, has been widely investigated in cervical cancer due to its downregulation in cell cycle [8]. Impaired *P16^INK4a^* gene function caused by promoter hypermethylation could result in uncontrolled cell proliferation and eventually oncogenesis [9–11]. In 1999, Wong et al. first reported that *P16^INK4a^* promoter hypermethylation was correlated with the advanced stage of CC [11]. Thereafter, numerous studies were carried out to assess the associations of *P16^INK4a^* hypermethylation with the development of SIL and CC. However, most of these studies only included relatively small sample size, leading to inconsistent results and a broad range of *P16^INK4a^* hypermethylation rates (from 2% to 93%) in cancer tissues [12, 13]. Moreover, the effect of *P16^INK4a^* promoter hypermethylation on different phases of cervical carcinogenesis (from LSIL to CC) is less summarized. Thus, a meta-analysis was conducted to systematically appraise the associations of *P16^INK4a^* methylation status with LSIL, HSIL, CC and their clinicopathological features.

## RESULTS

### Study characteristics

**Figure 1:**
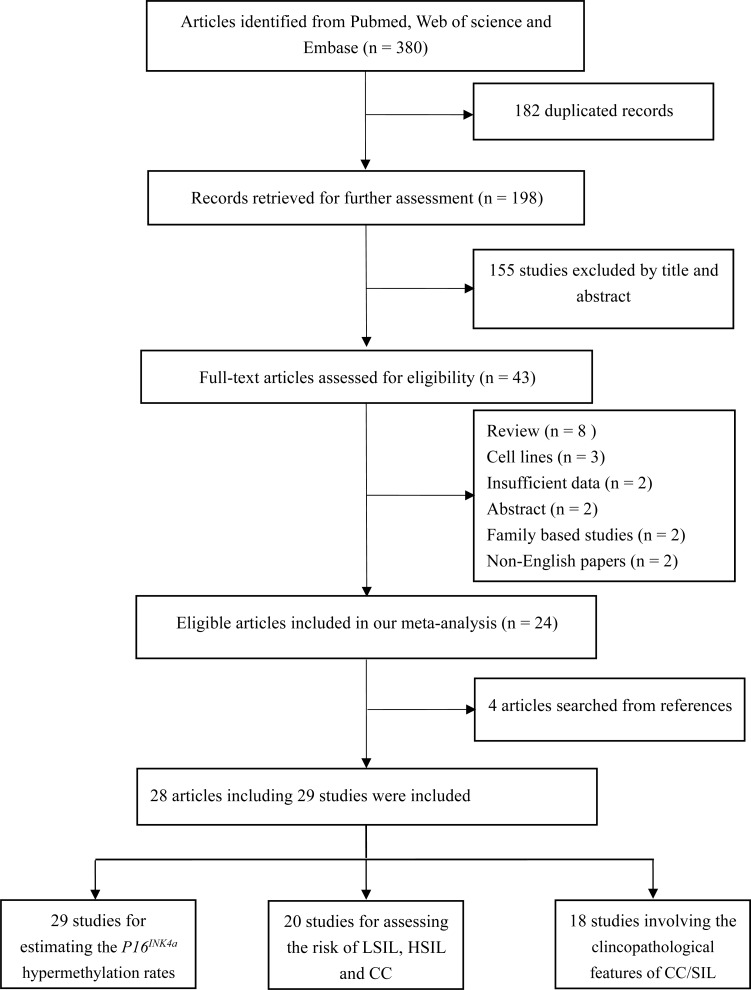
Flowchart for the study selection procedures in this meta-analysis

According to the definitions of the 2001 Bethesda System [14], LSIL encompassed cytopathic effects of HPV, mild dysplasia and cervical intraepithelial neoplasia (CIN) 1; HSIL contained moderate or severe dysplasia, carcinoma *in situ* (CIS) and CIN 2 or 3; CC encompassed squamous cell carcinoma (SCC) and adenocarcinoma (AdC). Based on these definitions, 43 articles were initially selected. Then, 19 articles were excluded due to *in vitro* experiments (*n* = 3), family-based designs (*n* = 2), abstracts (*n* = 2) or reviews (*n* = 8), non-English papers (*n* = 2) and insufficient data (*n* = 2). Manual search of references cited in the published articles identified four additional articles [15–18]. One article [19] contained data from two independent studies. Hence, 28 articles with 29 studies were finally included [11–13, 15–39]. Among these studies, all studies were eligible to estimate the *P16^INK4a^* hypermethylation rates; 20 studies (1 cross-sectional [13] and 19 case-control designs [16, 17, 19, 21–28, 30–35, 37, 38]) investigated the associations of *P16^INK4a^* methylation status with the risk of LSIL, HSIL and CC; 1254 SIL/CC patients from 18 studies (11 case-control studies [19, 21, 23, 25, 26, 31, 32, 35–38] and 7 case-only studies [11, 12, 15, 18, 20, 29, 39]) were eligible to assess the associations between *P16^INK4a^* methylation status and clinicopathological features. For most of these studies (26 studies), the methylation detection was based on methylation-specific PCR (MSP) (including MSP, nested MSP and MSP with another method (sequencing, prosequencing and BSP) for quality control). Only one study used plasma samples to detect methylation status [19]; other studies involved cervical tissues. Fifteen studies were conducted on Asians, 9 studies on Caucasians, 5 studies on other ethnicities (Brazilians, Moroccans and Senegalese). The flowchart for the study selection procedure was shown in Figure 1. The characteristics of included studies were summarized in Table 1.

**Table 1:** Characteristics of included studies in this meta-analysis

No.	First author (Year)	Country	Ethnicity	Study design	Sample size				Methylation detection method	Materials	Source of controls	Involved clinicopathological features	Quality scores
					Control	CC	HSIL	LSIL					
1	Nakashima 1999 [20]	Japan	Asian	Case-only	-	33	-	-	MSRE	Tissue	-	Tumor type	12
2	Wong 1999 [11]	China	Asian	Case-only	-	98	-	-	MSP	Tissue	-	FIGO stage, tumor grade, type	10
3	Dong 2001 [21]	Korea	Asian	Case-control	24	53	-	-	MSP and sequencing	Tissue	B	Tumor grade, type, early age	15
4	Virmani 2001 [22]	USA	Caucasian	Case-control	22	19	17	37	MSP	Tissue	H	-	13
5	Tsuda 2003 [15]	Japan	Asian	Case-only	-	53	33	9	MSP	Tissue	B	HPV infection	13
6	Gustafson 2004 [16]	USA	Caucasian	Case-control	11	-	17	11	Nested MSP	Tissue	H	-	11
7	Lea 2004 [23]	USA	Caucasian	Case-control	78	60	30	-	MSP	Tissue	H	FIGO stage, tumor grade, type, smoking, HPV infection	14
8	Yang tissue 2004 [19]	China	Asian	Case-control	100	85	-	-	MSP and sequencing	Tissue	A	FIGO stage, tumor grade, type	13
9	Yang plasma 2004 [19]	China	Asian	Case-control	30	40	-	-	MSP and sequencing	Plasma	H	-	13
10	Feng 2005 [17]	Senegal	African	Case-control	142	92	46	39	MSP	Tissue	M	-	10
11	Kim 2005 [24]	Korea	Asian	Case-control	11	41	19	11	MSP	Tissue	B	-	11
12	Lin 2005 [25]	Korea	Asian	Case-control	20	47	10	20	MSP	Tissue	B	Tumor type	11
13	Jeong 2006 [26]	Korea	Asian	Case-control	24	78	-	-	MSP	Tissue	B	FIGO stage, tumor type, early age, smoking	15
14	Kang 2006 [27]	Korea	Asian	Case-control	5	43	7	31	MSP and pyrosequencing	Tissue	B	-	13
15	Kekeeva 2006 [28]	Russia	Caucasian	Case-control	35	-	42	-	MSP	Tissue	H	-	10
16	Yang 2006 [29]	China	Asian	Case-only	-	127	-	-	MSP and sequencing	Tissue	-	FIGO stage, tumor grade, type	12
17	Ivanova 2006 [30]	Russia	Caucasian	Case-control	14	26	-	-	MSP and BSP	Tissue	A	-	11
18	Nehls 2008 [18]	Germany	Caucasian	Case-only	-	70	16	8	Nested BSM-PCR	Tissue	-	HPV infection	12
19	Attaleb 2009 [31]	Morocco	African	Case-control	20	22	-	-	MSP	Tissue	H	FIGO stage, tumor grade, HPV infection, early age	12
20	Furtado 2010 [32]	Brazil	Brazilian	Case-control	20	-	27	-	MSP	Tissue	H	HPV infection	11
21	Kim 2010 [33]	Korea	Asian	Case-control	41	69	67	32	Nested MSP	Tissue	B	-	13
22	Huang 2011 [34]	China	Asian	Case-control	15	26	49	23	MSP	Tissue	H	-	12
23	Lof-Ohlin 2011 [12]	Sweden	Caucasian	Case-only	-	109	-	-	Pyrosequencing	Tissue	-	-	11
24	Spathis 2011 [35]	Greece	Caucasian	Case-control	41	12	85	121	MSP	Tissue	H	Tumor type	12
25	Jha 2012 [36]	India	Asian	Case-control	100	125	-	-	MSP	Tissue	M	Smoking	12
26	Carestiato 2013 [13]	Brazil	Brazilian	Cross-sectional	28	29	49	35	MSP	Tissue	H	-	10
27	Banzai 2014 [37]	Japan	Asian	Case-control	24	53	22	-	MSP	Tissue	H	Tumor type	10
28	Blanco-Luquin 2015 [38]	Spain	Caucasian	Case-control	13	67	85	10	MSP	Tissue	H	Tumor type	15
29	Silveria 2015 [39]	Brazil	Brazilian	Cohort	-	40	-	-	MSP	Tissue	-	HPV infection	14

Abbreviation: CC, cervical cancer; LSIL, low-grade squamous intra-epithelial lesion; HSIL, high-grade squamous intra-epithelial lesion; MSRE, methylation-sensitive restriction endonucleases; MSP, methylation-specific PCR; BSP, bisculfite sequencing PCR; H, healthy controls; B, controls with benign gynecological diseases; A, autologous controls; M, mixed controls.

### Pooled rates of *P16*^INK4a^ hypermethylation in patients with LSIL, HSIL and CC

A total of 388 LSIL [13, 15–18, 22, 24, 25, 27, 33–35, 38, 39], 636 HSIL [13, 15–18, 22–25, 27, 28, 32–35, 37, 38] and 1439 CC [11–13, 15, 17–26, 29–31, 33–38] specimens were included in this meta-analysis. As summarized in Table 2, the pooled rates of *P16^INK4a^* hypermethylation showed an increasing trend (*p* < 0.001 for the differences in pooled rates) from LSIL tissues (21.4%, 95% confidence interval (CI): 15.0-29.7%) to HSIL tissues (30.9%, 95% CI: 21.9-41.7%) and ultimately to CC specimens (35.0%, 95%CI: 27.6-43.3%). The respective *P16^INK4a^* hypermethylation rates for Asians and Caucasians were similar: 24.6% and 21.5% in LSIL tissues; 31.9% and 27.2% in HSIL tissues; 33.7% and 38.2% in CC specimens. In CC specimens, the pooled rates did not significantly change after excluding one study using plasma samples (35.6%, 95% CI: 28.0-44.1%).

**Table 2:** Pooled hypermethylation rates of P16^*INK4a*^ in LSIL, HSIL and CC specimens

Comparison	Studies (N)	Specimens (N)	Heterogeneity		Model ^a^	Methylation rates (%)
			I^2^(%)	P_Q-test_		
LSIL						
Total	14	388	47	0.025	R	21.4 (15.0-29.7)
Asian	6	86	21	0.278	F	24.6 (16.1-35.5)
Caucasian	5	193	67	0.016	R	21.5 (9.8-41.0)
Others	3	109	59	0.088	R	13.8 (5.1-31.9)
HSIL						
Total	17	636	82	< 0.001	R	30.9 (21.9-41.7)
Asian	7	231	81	< 0.001	R	31.9 (18.2-49.7)
Caucasian	7	286	76	< 0.001	R	27.2 (16.6-41.2)
Others	3	119	88	< 0.001	R	34.5 (9.9-71.6)
CC						
Total	24	1439	88	< 0.001	R	35.0 (27.6-43.3)
Asian	14	941	87	< 0.001	R	33.7 (25.5-43.3)
Caucasian	6	363	85	0.006	R	38.2 (27.1-50.6)
Others	3	135	96	< 0.001	R	39.7 (26.7-54.3)

a When significant heterogeneity was found (I^2^≥ 50% or P_Q-test_ ≤ 0.1), the random-effects model (DerSimonian-Laird method) was used to pool the results; otherwise, the fixed-effects model (Mantel-Haenszel method) was applied.

Abbreviations: N, number; LSIL, low-grade squamous intra-epithelial lesion; HSIL, high-grade squamous intra-epithelial lesion; CC, cervical cancer; R, random-effects model.

### Association of *P16*^INK4a^ methylation status with LSIL risk

**Figure 2:**
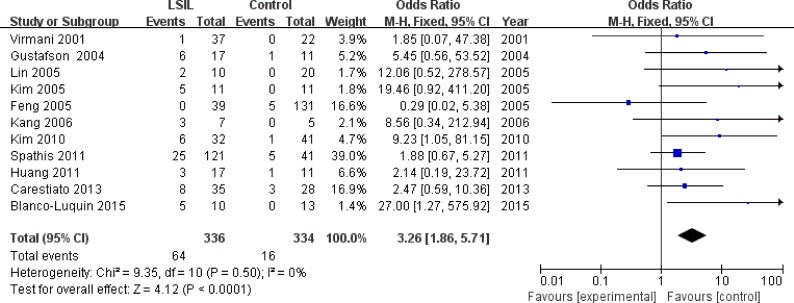
Forest plot for the association between *P16*^*INK4a*^ promoter hypermethylation and LSIL risk

Eleven studies [13, 16, 17, 22, 24, 25, 27, 33–35, 38], involving 336 LSIL patients and 334 controls, were included to assess the association between *P16^INK4a^* methylation status and LSIL risk. Overall, *P16^INK4a^* promoter hypermethylation was associated with a 3.26-fold (95% CI: 1.86-5.71, *p* < 0.001) increased risk of LSIL (Figure 2 and Table 3). This association remained significant in almost all subgroups, except for the “other ethnicities” subgroup (Table 3). No significant heterogeneity was found in all comparisons (I^2^: 0-42%).

**Table 3:** Pooled results for the association between ^*P16INK4a*^ promoter hypermethylation and LSIL risk

Comparisons	Studies (N)	Sample size (LSIL/controls)	Heterogeneity		Model ^a^	Effect size	
			I^2^(%)	P_Q-test_		OR (95% CI)	*P*
Total	11	336/334	0	0.499	F	3.26 (1.86-5.71)	< 0.001
Ethnicity							
Asian	5	77/88	0	0.817	F	7.76 (2.39-25.15)	0.001
Caucasian	4	185/87	4	0.374	F	2.98 (1.29-6.91)	0.011
Other ethnicities	2	74/159	42	0.190	F	1.39 (0.45-4.27)	0.565
Source of controls							
Healthy	6	237/126	0	0.677	F	2.79 (1.39-5.57)	0.004
Non-healthy^b^	5	99/208	23	0.266	F	4.52 (1.78-11.47)	0.001
Quality of studies							
High (≥ 12)	6	224/133	0	0.489	F	3.37 (1.58-7.21)	0.002
Low (< 12)	5	112/201	20	0.290	F	3.09 (1.35-7.09)	0.008

a When significant heterogeneity was found (I^2^≥ 50% or P_Q-test_ ≤ 0.1), the random-effects model (DerSimonian-Laird method) was used to pool the results; otherwise, the fixed-effects model (Mantel-Haenszel method) was applied.

b Non-healthy controls included autologous controls (normal tissues adjacent to LSIL specimens), controls with benign gynecological diseases and mixed controls.

Abbreviations: N, number; LSIL, low-grade squamous intra-epithelial lesion; F, fixed-effects model.

### Association of *P16*^INK4a^ methylation status with HSIL risk

**Figure 3:**
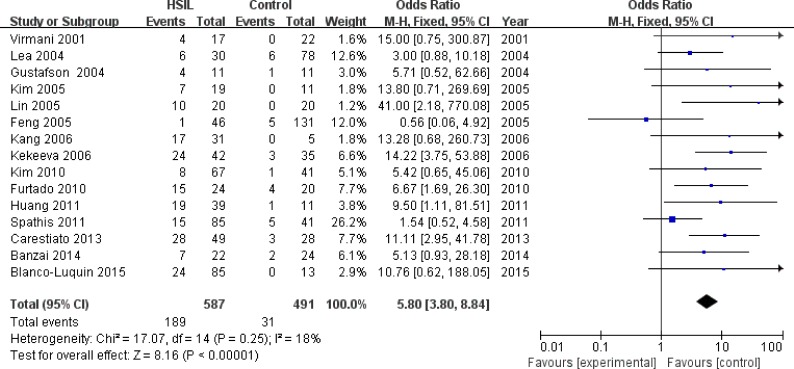
Forest plot for the association between *P16*^*INK4a*^ promoter hypermethylation and HSIL risk

Fifteen studies [13, 16, 17, 22–25, 27, 28, 32–35, 37, 38] with 587 HSIL patients and 491 controls were eligible to evaluate the association of *P16^INK4a^* methylation status with HSIL risk. A significant association was found between *P16^INK4a^* promoter hypermethylation and increased HSIL risk, with an odds ratio (OR) of 5.80 (95% CI: 3.80-8.84) and a *p* value of < 0.001 (Figure 3 and Table 4). This association remained significant in all subgroups (Table 4). We did not find significant heterogeneity in all comparisons (I^2^: 0-43%).

**Table 4:** Pooled results for the association between *P16*^*INK4a*^ promoter hypermethylation and HSIL risk

Comparisons	Studies (N)	Sample size (HSIL/controls)	Heterogeneity		Model ^a^	Effect size	
			I^2^(%)	P_Q-test_		OR (95% CI)	*P*
Total	15	587/491	18	0.253	F	5.80 (3.80-8.84)	< 0.001
Ethnicity							
Asian	6	198/112	0	0.869	F	9.70 (3.85-24.42)	< 0.001
Caucasian	6	270/200	38	0.374	F	4.61 (2.50-8.52)	< 0.001
Other ethnicities	3	119/179	43	0.167	F	5.25 (2.46-11.18)	< 0.001
Source of controls							
Healthy	9	393/272	22	0.247	F	5.74 (3.51-9.36)	< 0.001
Non-healthy ^b^	6	194/219	27	0.236	F	5.99 (2.61-13.74)	< 0.001
Quality of studies							
High (≥ 12)	7	354/211	0	0.453	F	4.08 (2.16-7.73)	< 0.001
Low (< 12)	8	233/280	17	0.298	F	7.80 (4.47-13.62)	< 0.001

a When significant heterogeneity was found (I^2^≥ 50% or P_Q-test_ ≤ 0.1), the random-effects model (DerSimonian-Laird method) was used to pool the results; otherwise, the fixed-effects model (Mantel-Haenszel method) was applied.

b Non-healthy controls included autologous controls (normal tissues adjacent to HSIL specimens), controls with benign gynecological diseases and mixed controls.

Abbreviations: N, number; HSIL, high-grade squamous intra-epithelial lesion; F, fixed-effects model.

### Association of *P16*^INK4a^ methylation status with CC risk

**Figure 4:**
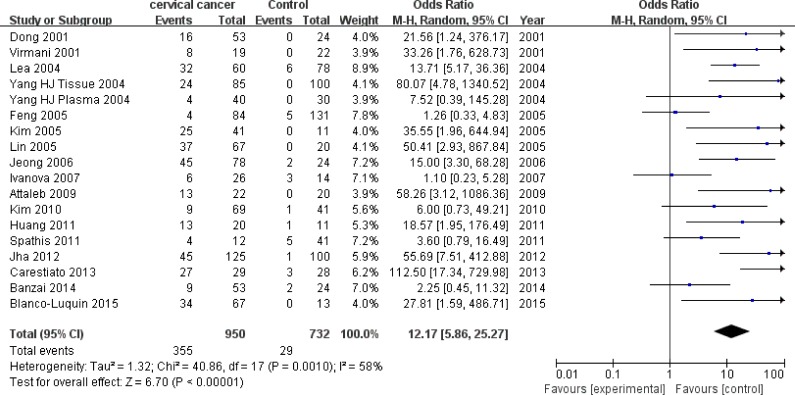
Forest plot for the association between *P16^INK4a^* promoter hypermethylation and CC risk

Eighteen studies [13, 17, 19, 21–26, 30, 31, 33–38] with 950 CC patients and 732 controls were included to appraise the effect of *P16^INK4a^* promoter hypermethylation on CC risk. There was a significant association between *P16^INK4a^* promoter hypermethylation and increased CC risk, with an OR of 12.17 (95% CI: 5.86-25.27) and a *p* value of < 0.001 (Figure 4 and Table 5). Consistent with the increasing rates of *P16^INK4a^* hypermethylation in LSIL, HSIL and CC specimens, we also found an increasing trend (*p* < 0.001) in effects of *P16^INK4a^* promoter hypermethylation on the risk of LSIL (OR = 3.26), HSIL (OR = 5.80) and CC (OR = 12.17).

**Table 5:** Pooled results for the association between *P16^INK4a^* promoter hypermethylation and CC risk

Comparisons	Studies (N)	Sample size (CC/controls)	Heterogeneity		Model ^a^	Effect size	
			I^2^(%)	P_Q-test_		OR (95% CI)	*P*
Total	18	950/732	58	0.001	R	12.17 (5.86-25.27)	< 0.001
Ethnicity							
Asian	10	631/385	19	0.272	F	18.94 (9.75-36.81)	< 0.001
Caucasian	5	270/200	60	0.039	R	6.83 (1.98-23.55)	0.002
Other ethnicities	3	135/179	88	< 0.001	R	9.87 (4.45-21.90)	< 0.001
Source of controls							
Healthy	9	322/267	44	0.073	R	13.67 (5.64-33.10)	< 0.001
Non-healthy	9	628/465	69	0.001	R	11.32 (3.28-39.05)	< 0.001
Quality of studies							
High (≥ 12)	11	583/491	0	0.495	F	18.81 (10.84-32.63)	< 0.001
Low (< 12)	7	427/311	77	< 0.001	R	8.83 (1.85-42.11)	0.006

a When significant heterogeneity was found (I^2^≥ 50% or P_Q-test_ ≤ 0.1), the random-effects model (DerSimonian-Laird method) was used to pool the results; otherwise, the fixed-effects model (Mantel-Haenszel method) was applied.

b Non-healthy controls included autologous controls (normal tissues adjacent to HSIL specimens), controls with benign gynecological diseases and mixed controls.

Abbreviations: N, number; CC, cervical cancer; R, random-effects model; F, fixed-effects model.

Since moderate heterogeneity was observed in the overall comparison (I^2^ = 58%), subgroup, meta-regression and Galbraith plot analyses were performed to seek the potential sources of heterogeneity. In subgroup analyses, *P16^INK4a^* promoter hypermethylation was consistently associated with increased CC risk in all subgroups (Table 5). However, moderate heterogeneity remained in most of the subgroups, except for the subgroups involving high-quality studies (I^2^ = 0%), Asians (I^2^ = 19%) and healthy controls (I^2^ = 44%). The results of meta-regression analyses indicated that ethnicity (*p* = 0.668), source of controls (*p* = 0.678) and quality of studies (*p* = 0.289) were not major sources of heterogeneity (Supplementary Table 1). The subsequent Galbraith plot depicted three outliers [13, 17, 30] as the potential origins of heterogeneity (Supplementary Figure 1). When we excluded these three studies, the association between *P16^INK4a^* methylation status and CC risk remained significant (OR = 17.36, 95% CI: 10.61-28.42, *p* < 0.001), followed by an effective reduction in I^2^ value from 58% to 12%.

### Association of *P16*^INK4a^ methylation status with clinicopathological features of SIL/CC

**Figure 5:**
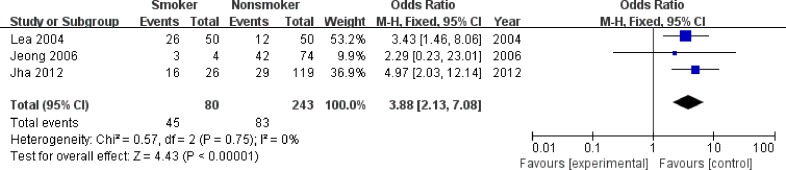
Forest plot for the association between *P16^INK4a^* promoter hypermethylation and smoking habit

We first evaluated the associations of *P16^INK4a^* methylation status with several risk factors for SIL/CC, including HPV infection (Positive *vs* Negative), smoking habit (Smoker *vs* Nonsmoker) and early age at diagnosis (< 50 *vs* ≥ 50) (Table 6), and observed that *P16^INK4a^* promoter hypermethylation was significantly associated with smoking habit, (OR = 3.88, 95% CI: 2.13-7.08, *P* < 0.001) (Figure 5), but was not correlated with HPV infection and early age at diagnosis (Supplementary Figure 2 and 3). In meta-analyses for the effects of *P16^INK4a^* methylation status on histological types (SCC *vs* AdC), clinical stages (FIGO stage: III + IV *vs* I + II) and tumor grades (Grade 2 + 3 *vs* Grade 1) in CC patients, no significant association was found (Table 6 and Supplementary Figure 4-6).

**Table 6:** Pooled results for the associations between *P16^INK4a^* hypermethylation and clinicopathological features of CC/SIL

Clinicopathological features	Studies (N)	Patients (N)	Heterogeneity		Model ^a^	Effect size	
			I^2^ (%)	P_Q-test_		OR (95% CI)	*P*
Risk factors for SIL/CC							
HPV infection (Positive vs Negative)	6	288	0	0.974	F	1.06 (0.49-2.28)	0.883
Smoking habit (Smoker vs Nonsmoker)	3	323	0	0.751	F	3.88 (2.13-7.08)	< 0.001
Early age at diagnosis (<50 vs ≥ 50)	3	153	0	0.380	F	0.91 (0.47-1.76)	0.774
Clinical and histological data of CC							
Tumor type (SCC vs AdC)	11	731	22	0.235	F	1.00 (0.68-1.48)	0.986
FIGO stage (III + IV vs I + II)	6	470	62	0.020	R	1.49 (0.62-3.56)	0.368
Tumor grade (G2 + G3 vs G1)	6	440	0	0.441	F	0.76 (0.46-1.24)	0.263

a When significant heterogeneity was found (I^2^≥ 50% or P_Q-test_ ≤ 0.1), the random-effects model (DerSimonian-Laird method) was used to pool the results; otherwise, the fixed-effects model (Mantel-Haenszel method) was applied.

Abbreviations: N, number; CC, cervical cancer; SCC, squamous cell carcinoma; AdC, adenocarcinoma; SIL, squamous intra-epithelial lesion; F, fixed-effects model ; R,random-effects model.

### Evidence grading

Because all eligible studies were observational, the Grading of Recommendations Assessment, Development and Evaluation (GRADE) process for all comparisons began as “low quality” [40]. For the comparisons of CC risk, HPV infection, early age at diagnosis, tumor type and clinical stage, the quality of evidence was further downgraded to “very low quality”, due to study limitations, inconsistency or imprecision (Supplementary Table 2).

### Sensitivity analyses for assessing the stability of pooled results

In all comparisons, sensitivity analyses by sequentially removing each study did not significantly change the pooled results, suggesting the stability of our meta-analyses (Supplementary Figure 7)

### Analyses for publication bias

In all comparisons, funnel plots did not reveal obvious asymmetry (Supplementary Figure 8). These observations, combined with the results of Egger's test (*p*_Egger_ > 0.05 for all comparisons), suggested that no significant publication bias was found.

## DISCUSSION

Previous studies have long aimed to seek methylation biomarkers associated with diagnosis, progression or prognosis of cervical neoplasia. Particularly, a bi-marker panel consisting of CADM1-M18 and MAL-M1 has been considered as a stable triage tool, which could be equally discriminatory for CIN3^+^ as cytology or cytology with HPV16/18 genotyping in HPV-positive women [41]. In contrast, although *P16^INK4a^* promoter hypermethylation has been linked to CC and SIL, the relatively small sample size of independent studies led to inconsistent results and a broad range of hypermethylation rates in cancer tissues. In this meta-analysis, on the basis of data from over 3000 subjects, we found that the hypermethylation rates in LSIL, HSIL and CC specimens were gradually increased, resulting in a growing trend in effects of *P16^INK4a^* hypermethylation on susceptibility to LSIL, HSIL and CC. These results, combined with the previous epidemiological evidence that *P16^INK4a^* hypermethylation was correlated with the progression of LSIL to HSIL [39, 42], suggest that *P16^INK4a^* promoter hypermethylation may be an epigenetic marker for the progression of cervical carcinogenesis. Hence, detecting *P16^INK4a^* hypermethylation may help clinicians to determine whether patients with cervical neoplasia are in disease regression, persistence or progression. Especially in patients with an initial diagnosis of LSIL, once *P16^INK4a^* hypermethylation is found, more effective clinical management for these patients are encouraged to conduct.

However, the existing evidence provides limited information on the prognostic value of *P16^INK4a^* hypermethylation in cervical neoplasia. In a case-series study from China, Yang et al. found no significant association between *P16^INK4a^* hypermethylation and overall survival [29]. In contrast, Blanco-Luquin et al. suggested that *P16^INK4a^* hypermethylation was correlated with improved disease-free survival [38]. Considering that these two studies involved relatively small sample sizes and inconsistent follow-up times, better designed studies are required to address this issue.

The interaction of *P16^INK4a^* hypermethylation with HPV infection is controversial in various HPV-related cancers. For HPV-related oral and oropharyngeal cancer (OSCC) [43], Schlecht et al. found four *P16^INK4a^*-specific CPG loci associated with HPV infection in OSCC tissues [44], while another study from Chile failed to replicate this association [45]. For cervical carcinoma, previous functional studies have suggested that *P16^INK4a^* promoter hypermethylation mainly occurred at early cervical tumor cell populations without HPV's E7 transcription [46]. In this meta-analysis, HPV infection was not associated with *P16^INK4a^* hypermethylation in patients with SIL/CC. *P16^INK4a^* hypermethylation was associated with a 3.26-fold increased risk of LSIL, suggesting the effect of *P16^INK4a^* hypermethylation on early stage of cervical oncogenesis. All these findings may suggest that *P16^INK4a^* hypermethylation is an early event in cervical carcinogenesis, independent of HPV infection,.

In this meta-analysis, smoking habit was associated with increased *P16^INK4a^* hypermethylation rates in patients with SIL/CC. The correlation between smoking habit and *P16^INK4a^* hypermethylation has been revealed in several cancers, including non-small cell lung cancer (NSCLC) and esophageal squamous cell carcinoma (ESCC) [47, 48]. In a longitudinal study, Ma et al. [49] reported that smoking initiation was associated with a 3.76-fold increased risk of the appearance of *P16^INK4a^* hypermethylation in normal cervical smears, providing direct evidence for the relationship between smoke exposure and subsequent acquisition of *P16^INK4a^* hypermethylation in cervix. As a well known risk factor for CC [50], exposure to tobacco smoke, or to its key ingredients (such as nicotine or its derivative), is followed by overexpression of DNA methyltransferases 1, 3A or 3B [51, 52], which has been reported to cause hypermethylation of *P16^INK4a^* promoter in mice and cancer patients [53]. Considering that our pooled results were based on the data from relatively few studies, more studies with large sample size are required to repeat this finding.

Moderate heterogeneity was found in our meta-analysis for the association between *P16^INK4a^* hypermethylation and CC risk. Therefore, the results were first pooled by using the random-effects model, which cautiously estimates the study weights after accounting for the inter-study differences [54]. Then, by depicting the Galbraith plot, we found that three studies might be the major contributors to the existence of heterogeneity [13, 17, 30]. Notably, the hypermethylation rates of CC tissues enormously varied across these three studies (from 5% [17] to 23% [30] and to 95% [13]), suggesting the existence of inter-study differences. By appraising these three studies using our quality scoring system, we found some common flaws for these studies, including lack of biospecimen information [13, 17, 30], lack of information on conventional risk factors [17, 30], and lack of quality controls for methylation detection [13, 17]. Otherwise, two of three studies collected non-healthy samples (autologous tissues and samples with atypical squamous cells) as their controls [17, 30]. All these issues may lead to the heterogeneous results. Thus, to increase the stability of results, subsequent association analyses for *P16^INK4a^* hypermethylation and CC risk should collect healthy controls, and provide adequate information on related confounding factors.

The following limitations merit consideration. First, most of included studies used the MSP method to detect *P16^INK4a^* methylation status. As a qualitative method, MSP mainly relies on primer designs to guarantee its accuracy [55]. However, the included studies applied different primers to detect methylation status, causing the potential bias that the promoter regions detected by MSP might not always be uniform. Second, lack of clinical data for each participant limited our ability to adjust for other covariates, such as age at primiparity and menopausal status. Finally, most of included studies adopted case-control or case-only design. This might lead to some selection bias due to inherent drawback of retrospective studies. Therefore, large prospective studies should be carried out with consistent primer designs, quantitative methylation analyses and multiple clinical data.

In this meta-analysis, *P16^INK4a^* hypermethylation rates showed an increasing trend from LSIL to HSIL and ultimately to CC, causing the increasing effects of *P16^INK4a^* hypermethylation on susceptibility to LSIL, HSIL and CC. Moreover, *P16^INK4a^* hypermethylation was also correlated with smoking habit in patients with CC/SIL. Future studies are warranted to repeat these findings and elucidate the underlying mechanism.

## MATERIALS AND METHODS

### Literature search

This meta-analysis was reported based on the PRISMA statement [56]. Electronic databases, including Pubmed, EMBASE and Web of Science (up to April 19, 2016), were searched by using the combinations of following terms: (*P16^INK4a^* or *P16* or *CDKN2A*) and (methylation or promoter methylation or DNA methylation) and (cervical cancer/cervical tumor/cervical neoplasia or SIL/LSIL/HSIL/or cervical dysplasia/CIN/CIS). Reference lists in reviews and retrieved articles were also checked for other relevant studies.

### Eligibility criteria

Eligible studies were required to meet the following criteria: (1) an observational design (cohort, case-control, case-only or cross-sectional studies); (2) studies assessing the associations of *P16^INK4a^* methylation status with LSIL, HSIL, CC or their clinicopathological features; (3) studies with sufficient data to calculate the hypermethylation rates, ORs and their 95% CI; (4) written in English.

Exclusion criteria were as follows: (1) reviews, letters, abstracts and case reports; (2) reports with insufficient data; (3) studies regarding *in vitro* or *ex vivo* experiments; (4) family-based studies; (5) studies focusing on benign gynecological diseases. For duplicated data, only the most recent or detailed data set was selected.

### Data extraction

According to a predefined data collection form, data extraction was carried out by two independent authors (XBW and YDH), with any discrepancies resolved by consensus. The following information for eligible studies was collected: the first author's name, publication year, study design, ethnicity (country), involved diseases (LSIL, HSIL or CC) or their clinicopathological features (tumor type, clinical stage and tumor grade; age at diagnosis, smoking habit and HPV status), sample size, methods for methylation detection, sample materials, source of controls, and quality of studies.

### Quality assessment of eligible studies

According to a predefined system derived from the REMARK [57, 58] and BRISQ [59] guidelines, the quality of eligible studies was appraised by two independent authors (NHC and SZ). This quality scoring system involved 18 items, allowing for assessment of study design, study population, biospecimen information, methylation detection, clinicopathological features and results analysis (Supplementary Table 3). Studies that reported at least 12 items were considered as high-quality studies.

### Evidence grading

Once data synthesis was complete, we used the GRADE process to rate the quality of evidence for each comparison as high, moderate, low or very low [40]. Each rating was mainly based on 8 factors, involving study limitations, inconsistency, indirectness, imprecision, reporting bias, magnitude of effect, dose-response gradient and handling of potential confounders [40] (appraised by XBW and NHC).

### Statistical Methods

The *P16^INK4a^* hypermethylation rates in LSIL, HSIL and CC specimens were estimated using the inverse variance method [60]. Pooled ORs and their 95% CIs were calculated to assess the associations of *P16^INK4a^* methylation status with LSIL, HSIL, CC and their clinicopathological features. The heterogeneity across the included studies was evaluated by the χ^2^-based Q-test and I^2^ statistic. I^2^ values of 25%, 50% and 75% were set as the cutoff values for mild, moderate and extensive heterogeneity, respectively [61]. When significant heterogeneity was found (I^2^ ≥ 50% or *P*_Q-test_ ≤ 0.1), the random-effects model (DerSimonian-Laird method) was used to pool the results; otherwise, the fixed-effects model (Mantel-Haenszel method) was applied. To further seek the potential sources of heterogeneity, meta-regression and subgroup analyses were performed based on ethnicity, source of controls and quality of studies. Then, a Galbraith plot was depicted to visualize the contribution of individual studies to the overall heterogeneity. To further appraise the stability of the pooled results, sensitivity analyses were performed by sequentially omitting each study or removing the outliers depicted by the Galbraith plot [62]. Publication bias was assessed qualitatively by funnel plots and quantitatively by the Egger's test [63]. An asymmetric funnel plot and *P*_Egger_ ≤ 0.05 suggested the existence of publication bias. All the above analyses were conducted by RevMan 5.2 (The Nordic Cochrane Centre, The Cochrane Collaboration) and STATA 12.0 (Stata, College, TX, USA).

## SUPPLEMENTARY MATERIAL, SUPPLEMENTARY TABLES


