# Supplementary material for: Associations of P16INK4a promoter hypermethylation with squamous intra-epithelial lesion, cervical cancer and their clinicopathological features: a meta-analysis
Source: Oncotarget. 2016 Sep 22;8(1):1871–83. doi: 10.18632/oncotarget.12202 (PMC5352104; doi:10.18632/oncotarget.12202)
Supplement: Supplementary file 1 [file oncotarget-08-1871-s001.pdf]

Associations of *P16*^{INK4a} promoter hypermethylation with squamous intra-epithelial lesion, cervical cancer and their clinicopathological features: a meta-analysis

Supplementary Material

No. of Supplementary Tables: 3; **No. of Supplementary Figures:** 8

Supplementary Tables

Supplementary Table 1: The univariate meta-regression results of the association between *P16*^{INK4a} hypermethylation and CC risk.

Covariates	Coefficient	Standard error	P value	95% CI	τ^2 value (%)	I^2 res (%)
Ethnicity	-0.293	0.470	0.540	-1.280～0.694	-3.03	53.17
Source of controls	-0.273	0.714	0.707	-1.772～1.223	-6.23	54.02
Quality of studies	0.696	0.702	0.335	-0.779～2.171	6.03	49.42

Supplementary Table 2: GRADE assessment for the quality of evidence in this meta-analysis.

Comparison [a]	Quality assessment [b]								Summary of findings			Quality of evidence
									Study event rates		OR (95%CI)	
	Limitations	Inconsistency	Indirectness	Imprecision	Reporting bias	Magnitude of effect	Dose-response gradient	Potential confounders [g]	Controls	Cases		
LSIL risk	Serious [c]	No serious	No serious	No serious	Undetected	Large [f]	No	No	16/334 (4.8%)	64/336 (19.0%)	3.26 (1.86-5.71)	Low
HSIL risk	Serious [c]	No serious	No serious	No serious	Undetected	Large [f]	No	No	31/491 (6.3%)	189/587 (32.2)	5.80 (3.80-8.84)	Low
CC risk	Serious [c]	Moderate [d]	No serious	No serious	Undetected	Large [f]	No	No	29/732 (4.0%)	355/950 (37.4%)	12.17 (5.86-25.27)	Very low
HPV infection	No serious	No serious	No serious	Serious [e]	Undetected	No	No	No	54/79 (68.4%)	71/174 (40.8%)	1.06 (0.49-2.28)	Very low
Smoking	No serious	No serious	No serious	No serious	Undetected	Large [f]	No	No	83/243 (34.2%)	45/80 (56.3%)	3.88 (2.13-7.08)	Low
Early age	No serious	No serious	No serious	Serious [e]	Undetected	No	No	No	33/66 (50.0%)	41/87 (47.1%)	0.91 (0.47-1.76)	Very low
Tumor type	Serious [c]	No serious	No serious	No serious	Undetected	No	No	No	64/165 (38.8%)	214/566 (37.8%)	1.00 (0.68-1.48)	Very low
FIGO stage	No serious	Moderate [d]	No serious	No Serious	Undetected	No	No	No	120/336 (35.7%)	57/134 (42.5%)	1.49 (0.62-3.56)	Very low
Tumor grade	No serious	No serious	No serious	No Serious	Undetected	No	No	No	36/105 (34.3%)	110/335 (32.8%)	0.76 (0.46-1.24)	Low

[a] Because all eligible studies were observational, the GRADE assessment for all comparisons began as "low quality".

[b] The quality of evidence for each comparison can be downgraded due to study limitation, imprecision, inconsistency, indirectness and publication bias, and can be rated up because of a large magnitude of effect size, a dose-response gradient or presence of potential confounders.

[c] Limitations for no reasonable criteria for control selection, inconsistent primers for methylation detection, or no match for demographics between cases and controls.

[d] Inconsistency due to moderate heterogeneity among included studies (I^2 = 58% for CC risk; I^2 = 62% for FIGO stage).

[e] Imprecision due to small sample size with a total number of participants fewer than 300.

[f] Large magnitude of effect (OR > 2) with no plausible confounders.

[g] When all plausible residual confounders or biases would increase our confidence in the estimated effect, the quality of evidence can be rated up.

Abbreviation: LSIL, low-grade squamous intra-epithelial lesion; HSIL, high-grade squamous intra-epithelial lesion; CC, cervical cancer.

Supplementary Table 3: Definitions of 18 items in our quality scoring system.

**Study design**
1.Study objective definition: state the study objectives, prespecified hypothesis or study protocol
2. Sample size: state a statistical sample size or power calculation
3. Population source: state health care setting from which patients were recruited
4. Population selection criteria: state inclusion or exclusion criteria
5. Population demographic characteristics: state the population demographic characteristics (e.g., age, age ate primiparity and menopausal status)
6. Diagnosis of patients: state the criteria or guidelines to diagnose the included patients
**Biospecimen information**
1. Biospecimen characteristics: state biospecimen type and anatmical site
2. Biospecimen management: state the methods of collection and storage
**Methylation detection**
1. Assay method: state the type of assay method used to detect methylation status (MSP, BSP or pyrosequencing, etc)
2. Primer designs: state the primer sequences
3. Quality control: state the method of quality control
4. Blinding of laboratory staff: methylation detection done under "blinded" condition
**Clinicopathological features**
1. Clinical and pathological data: state the clinical and pathological data (such as tumor type, stage and grade)
2. Conventional risk factors: state the conventional risk factors (such as HPV infection, smoking habit)
3. Other biomarkers: state other biomarker relating with disease (such as methylation status of other genes, point mutation and expression level)
**Results analysis**
1. Univariate estimate: report the effect of methylation status on outcome
2. Multivariate estimate: adjusted for risk factors or other biomarkers
3. Missing data: state the number of patients with missing data and how to deal with it

Supplementary Figures

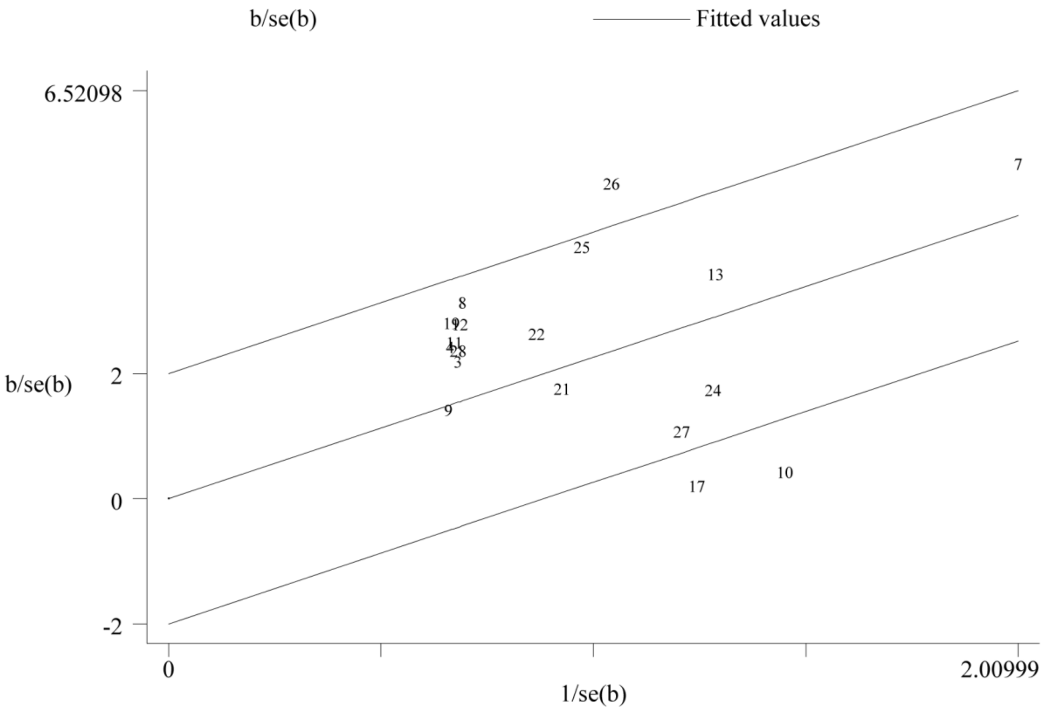

Supplementary Figure 1: Galbraith plot for the association between *P16*^{INK4a} promoter hypermethylation and CC risk. Each number is the number of the respective study included into our meta-analysis (shown in Table 1).

Study or Subgroup	Positive Events	Positive Total	Negative Events	Negative Total	Weight	Odds Ratio M-H, Random, 95% CI	Year
Tsuda 2003	8	9	39	44	11.2%	1.03 [0.11, 10.01]	2003
Lea 2004	29	53	3	7	22.9%	1.61 [0.33, 7.91]	2004
Nehls 2008	15	81	3	13	29.4%	0.76 [0.19, 3.09]	2008
Attaleb 2009	9	15	4	7	17.6%	1.13 [0.18, 6.93]	2009
Furtado 2010	10	16	5	8	18.9%	1.00 [0.17, 5.77]	2010
Silveira 2015	5	35	0	0		Not estimable	2015
**Total (95% CI)**		**209**		**79**	**100.0%**	**1.05 [0.49, 2.26]**	
Total events	76		54				

Heterogeneity: Tau² = 0.00; Chi² = 0.49, df = 4 (P = 0.97); I² = 0%
Test for overall effect: Z = 0.13 (P = 0.89)

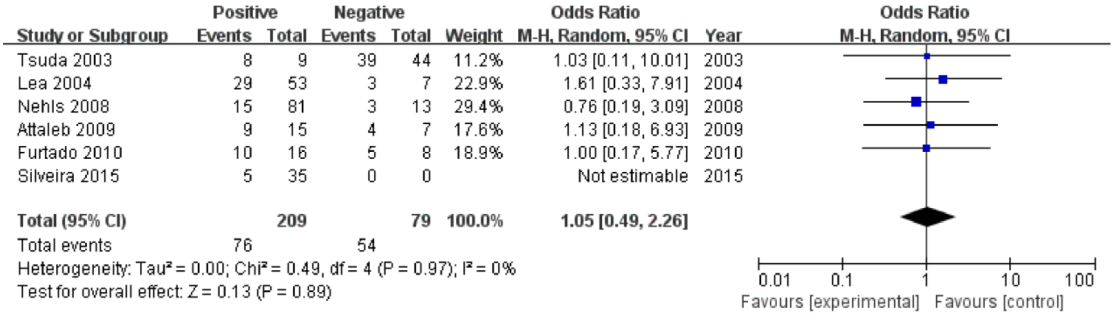

Supplementary Figure 2: Forest plot for the association between *P16*^{INK4a} promoter hypermethylation and HPV infection.

Study or Subgroup	≤ 50 years Events	≤ 50 years Total	> 50 years Events	> 50 years Total	Weight	Odds Ratio M-H, Fixed, 95% CI	Year
Dong 2001	11	31	5	22	20.6%	1.87 [0.54, 6.46]	2001
Jeong 2006	23	43	22	35	61.6%	0.68 [0.27, 1.69]	2006
Attaleb 2009	7	13	6	9	17.9%	0.58 [0.10, 3.40]	2009
**Total (95% CI)**		**87**		**66**	**100.0%**	**0.91 [0.47, 1.76]**	
Total events	41		33				

Heterogeneity: Chi² = 1.94, df = 2 (P = 0.38); I² = 0%
Test for overall effect: Z = 0.29 (P = 0.77)

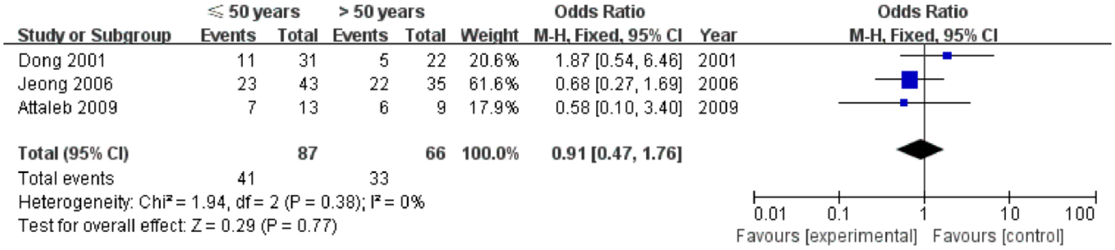

Supplementary Figure 3: Forest plot for the association between *P16*^{INK4a} promoter hypermethylation and early age at diagnosis.

Study or Subgroup	SCC Events	SCC Total	AdC Events	AdC Total	Weight	Odds Ratio M-H, Fixed, 95% CI	Year
Wong 1999	29	90	2	7	5.0%	1.19 [0.22, 6.50]	1999
Nakashima 1999	1	27	0	5	1.5%	0.62 [0.02, 17.39]	1999
Dong 2001	12	31	4	22	5.7%	2.84 [0.77, 10.45]	2001
Lea 2004	25	41	7	19	7.4%	2.68 [0.87, 8.24]	2004
Yang HJ Tissue 2004	18	61	6	24	12.1%	1.26 [0.43, 3.68]	2004
Lin 2005	33	47	17	20	14.1%	0.42 [0.10, 1.65]	2005
Jeong 2006	36	66	9	12	13.8%	0.40 [0.10, 1.61]	2006
Yang 2006	24	98	8	29	18.5%	0.85 [0.33, 2.17]	2006
Spathis 2011	1	8	3	4	7.0%	0.05 [0.00, 1.04]	2011
Banzai 2014	7	42	2	11	5.3%	0.90 [0.16, 5.10]	2014
Blanco-Luquin 2015	28	55	6	12	9.6%	1.04 [0.30, 3.62]	2015
**Total (95% CI)**		**566**		**165**	**100.0%**	**1.00 [0.68, 1.48]**	
Total events	214		64				

Heterogeneity: Chi² = 12.80, df = 10 (P = 0.23); I² = 22%
Test for overall effect: Z = 0.02 (P = 0.99)

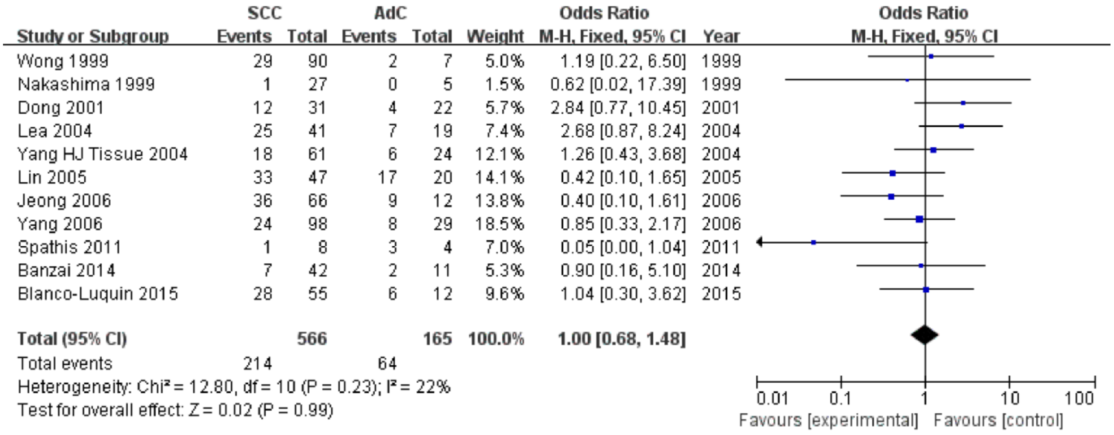

Supplementary Figure 4: Forest plot for the association between *P16*[*INK4a*] promoter hypermethylation and tumor type.

Study or Subgroup	Stage III + IV Events	Stage III + IV Total	Stage I + II Events	Stage I + II Total	Weight	Odds Ratio M-H, Random, 95% CI	Year
Wong 1999	14	24	17	74	21.0%	4.69 [1.77, 12.45]	1999
Lea 2004	19	30	13	30	20.4%	2.26 [0.80, 6.36]	2004
Yang HJ Tissue 2004	11	44	13	41	21.4%	0.72 [0.28, 1.85]	2004
Yang 2006	2	20	30	107	15.3%	0.29 [0.06, 1.30]	2006
Jeong 2006	2	3	43	75	8.8%	1.49 [0.13, 17.14]	2006
Attaleb 2009	9	13	4	9	13.2%	2.81 [0.48, 16.43]	2009
**Total (95% CI)**		**134**		**336**	**100.0%**	**1.49 [0.62, 3.56]**	
Total events	57		120				

Heterogeneity: Tau² = 0.69; Chi² = 13.33, df = 5 (P = 0.02); I² = 62%
Test for overall effect: Z = 0.90 (P = 0.37)

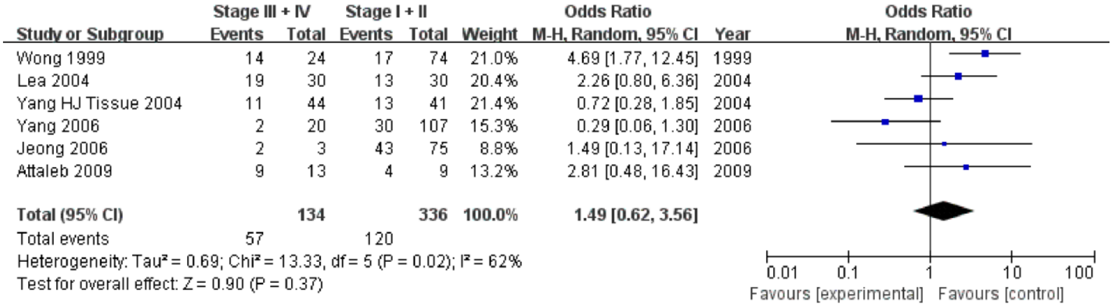

Supplementary Figure 5: Forest plot for the association between *P16*[*INK4a*] promoter hypermethylation and clinical stage of CC.

Study or Subgroup	G2-G3 Events	G2-G3 Total	G1 Events	G1 Total	Weight	Odds Ratio M-H, Fixed, 95% CI	Year
Wong 1999	27	80	4	18	12.1%	1.78 [0.53, 5.94]	1999
Dong 2001	14	45	2	8	6.5%	1.35 [0.24, 7.57]	2001
Lea 2004	31	58	1	2	2.5%	1.15 [0.07, 19.25]	2004
Yang HJ Tissue 2004	11	53	12	30	34.0%	0.39 [0.15, 1.05]	2004
Yang 2006	18	84	13	40	38.7%	0.57 [0.24, 1.32]	2006
Attaleb 2009	9	15	4	7	6.1%	1.13 [0.18, 6.93]	2009
**Total (95% CI)**		**335**		**105**	**100.0%**	**0.76 [0.46, 1.24]**	
Total events	110		36				

Heterogeneity: Chi² = 4.80, df = 5 (P = 0.44); I² = 0%
Test for overall effect: Z = 1.12 (P = 0.26)

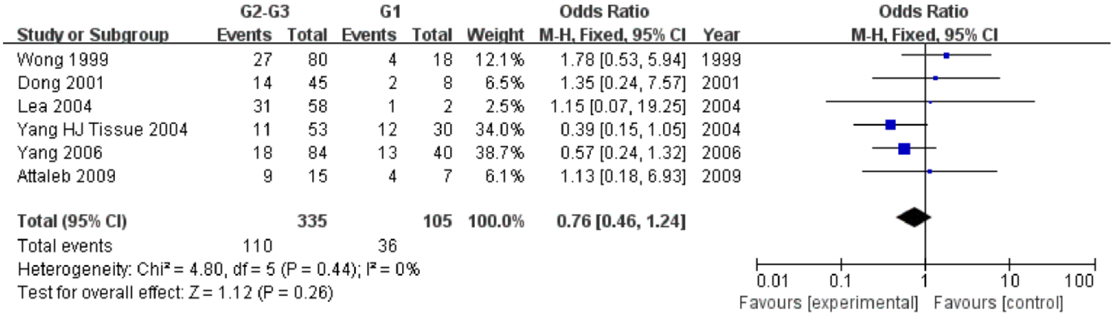

Supplementary Figure 6: Forest plot for the association between *P16*[*INK4a*] promoter hypermethylation and tumor grade.

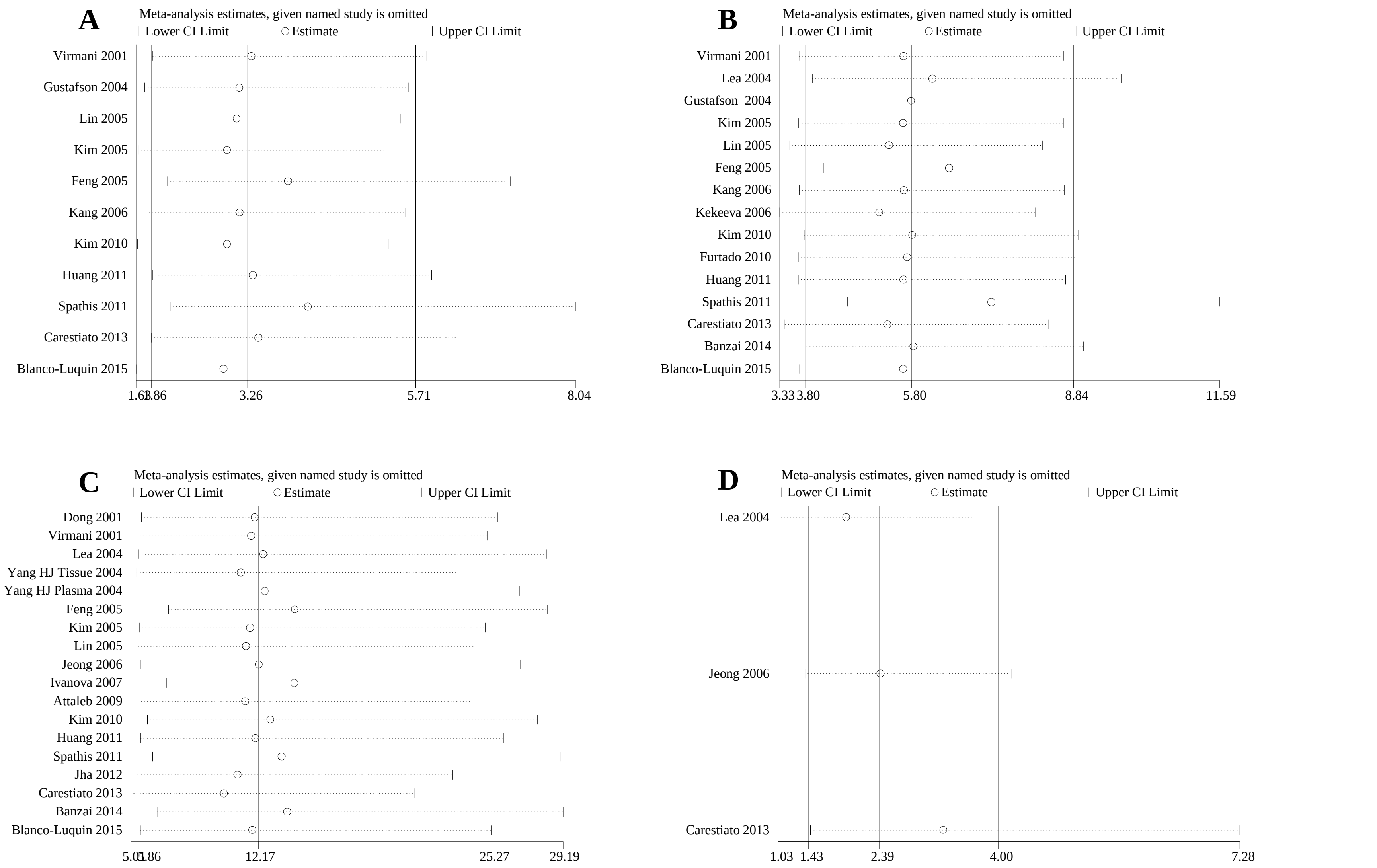

Supplementary Figure 7: Sensitivity analyses in this meta-analysis. (A) sensitivity analyses for the association between $P16^{INK4a}$ promoter hypermethylation and LSIL risk; (B) sensitivity analyses for the association between $P16^{INK4a}$ promoter hypermethylation and HSIL risk; (C) sensitivity analyses for the association between $P16^{INK4a}$ promoter hypermethylation and CC risk; (D) sensitivity analyses for the association between $P16^{INK4a}$ promoter hypermethylation and smoking habit. The results were computed by sequentially omitting each study. Bar represents 95% CI. The center of bars represents the summary effects when omitting corresponding studies.

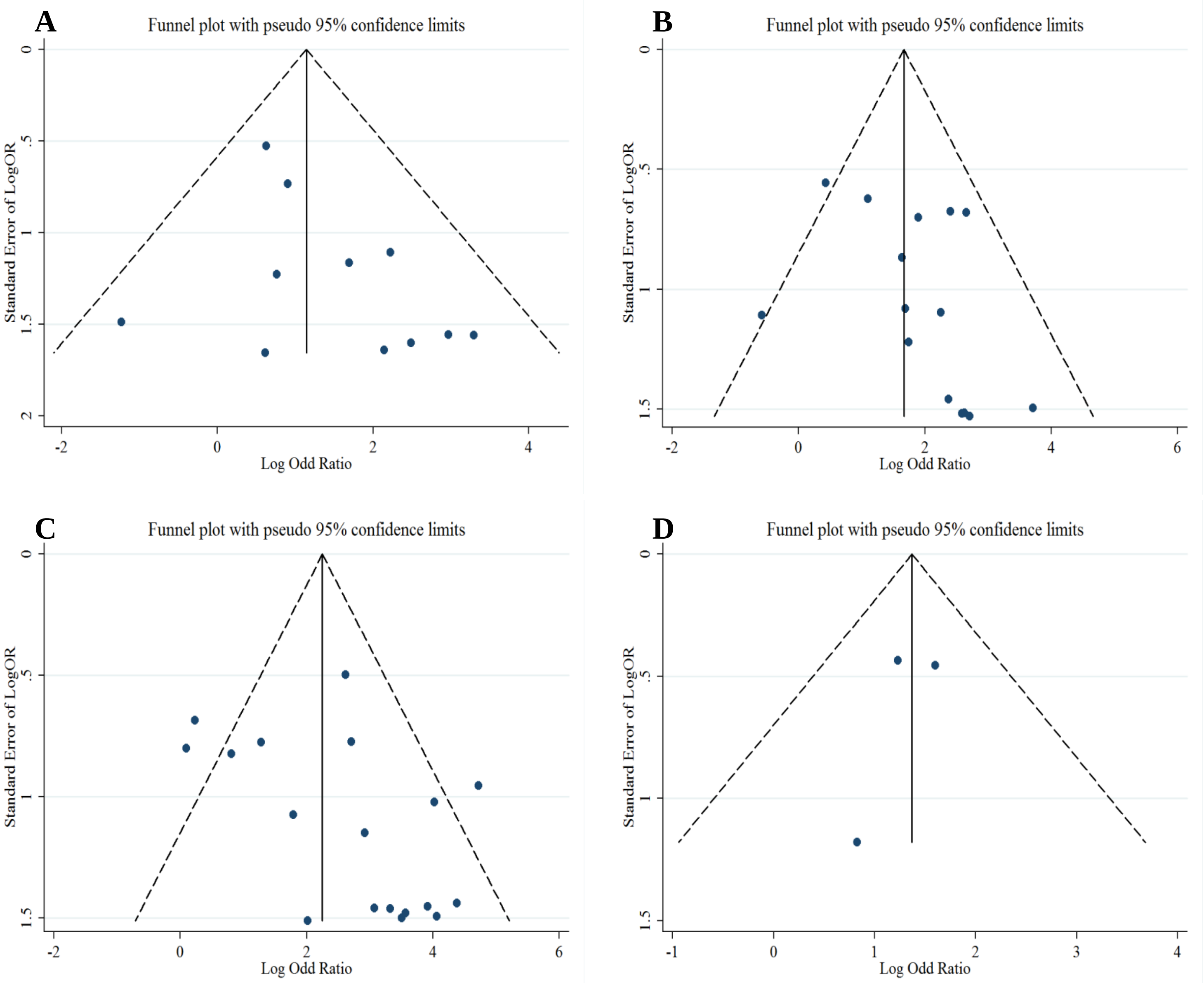

Supplementary Figure 8: Funnel plots in this meta-analysis. (A) funnel plot for the association between *P16*^{INK4a} promoter hypermethylation and LSIL risk; (B) funnel plot for the association between *P16*^{INK4a} promoter hypermethylation and HSIL risk; (C) funnel plot for the association between *P16*^{INK4a} promoter hypermethylation and CC risk; (D) funnel plot for the association between *P16*^{INK4a} promoter hypermethylation and smoking habit.
